# COMMENTARY ON: REDUCED PHYSICAL ACTIVITY LEVEL WAS ASSOCIATED WITH POORER QUALITY OF LIFE DURING THE EARLY PHASE OF THE COVID-19 PANDEMIC: A SUB-STUDY OF THE LAST-LONG TRIAL

**DOI:** 10.2340/jrm.v56.37556

**Published:** 2024-03-07

**Authors:** Josef FINSTERER

**Affiliations:** Neurology Department, Neurology & Neurophysiology Center, Postfach 20, AT-1180 Vienna, Austria

I read with interest Hokstad et al’s (1) article about a cross-sectional study on the impact of physical activity on quality of life (QoL) in 118 patients with a recent stroke during the pandemic. Compared with pre-pandemic times, 80 patients reported less physical activity, 50 reduced QoL, 43 reported poorer mental health, and 40 reported feelings of loneliness (1). Less physical activity was associated with reduced QoL (1). It was concluded that two-thirds of stroke patients reported reduced physical activity during the pandemic and lower physical activity was associated with reduced QoL (1). The study is impressive, but several points require discussion.

QoL of stroke patients depends heavily on the outcome of the stroke, on comorbidities, and the current medication. Therefore, it is necessary to know whether the modified Rankin scale (mRS) of the 118 included patients correlated with QoL. Did those with low mRS also have better QoL? Most likely, QoL was negatively correlated with the number of comorbidities.

Because stroke is often complicated by post-stroke depression, it is necessary to know how many were already taking antidepressants before the pandemic and how many were taking antidepressants following a stroke because of new-onset depression or exacerbation of a pre-existing mood disorder.

A limitation of the study is that it was conducted using an electronic questionnaire. Questionnaires have the disadvantage that it is difficult to assess whether the answers are correct and whether the patients themselves or a relative, caregiver, or friend was completing the form, It is also possible that different stroke patients, depending on their cognitive abilities, understand and answer questions differently.

Another limitation is that new illness or relapses of pre-existing illnesses between the stroke and the study were not considered causes of decreased QoL. It is necessary to know how many of the 118 patients had a further illness after the stroke and how many relapsed with an already known disease. Restricted mobility may not only be the result of a stroke, but can also be due to many other neurological, orthopaedic, cardiac, pulmonary, or psychiatric diseases.

Another limitation is that the vaccination status of the included patients was not reported. Since adequate immunization can provide a sense of security regarding not being re-infected, it would be useful to know how many of the patients were fully, partially or nor immunized against SARS-CoV-2.

Other questions are of interest. It is known that people with pets are usually more mobile than those without pets (2). Did the questionnaire also contain a question regarding the presence of a pet? Was having a pet associated with more mobility than those without a pet?

Mobility also depends heavily on whether a patient was engaged in physical activity before the stroke. Was there a difference in mobility after a stroke between those who exercised regularly before the stroke and those who did not exercise before the stroke?

In summary, the degree of mobility and therefore the QoL after a stroke depends on several factors, such as the severity of stroke, comorbidities (diseases that occur before and after the stroke), co-medications, presence of an affective psychiatric illness before or after the stroke, and exercise before the stroke. All of these factors should be included in the analysis of the impact of post-stroke mobility on QoL.
